# A societal challenge with high public health relevance: ADHD in children and adolescents in Germany

**DOI:** 10.25646/12675

**Published:** 2024-09-18

**Authors:** Robert Schlack, Marcel Romanos

**Affiliations:** 1 Robert Koch Institute, Department of Epidemiology and Health Monitoring, Berlin, Germany; 2 University Hospital Würzburg, Centre of Mental Health, Department of Child and Adolescent Psychiatry, Psychosomatics and Psychotherapy, Würzburg, Germany

Attention-deficit/hyperactivity disorder (ADHD) is one of the most common mental disorders of childhood and adolescence with potentially lifelong individual, family and social consequences and high public health relevance. The prevalence of diagnosed ADHD in Germany is estimated using different data sources: claims data from statutory health insurance funds (administrative data) and parent-reported diagnoses by physicians or psychologists from the German Health Interview and Examination Survey for Children and Adolescents (KiGGS) conducted by the Robert Koch Institute [1]. In the first decade of the millennium, increasing ADHD prevalence rates were reported from administrative data for Germany, while the frequency of parent-reported diagnoses remained unchanged or declined. This development was accompanied by controversies (‘fashion diagnosis of ADHD’), particularly with regard to possible over-, under- or misdiagnosis. However, neither administrative nor epidemiologically reported diagnostic data of ADHD was known to be clinically valid.

Differences in the ADHD prevalence rates are not only of academic interest. They indeed have practical implications for health policy and health care planning. Underestimating prevalence can mean that too few resources are allocated to help those affected. On the one hand, this can lead to latencies, inappropriate care, or a general lack of care, which can significantly impair the quality of life of affected children and their families and lead to problems in school, education and work. Overestimation, on the other hand, can lead to misallocation of resources.

Data-linkage studies with the possibility to directly compare administrative, epidemiological and clinical ADHD diagnostic data and the quality of care of ADHD patients have been repeatedly called for in order to clarify their actual needs and care situation. The consortium project ‘INTEGRATE-ADHD – Comparison and Integration of Administrative and Epidemiological ADHD Diagnostic Data by Clinical Assessment’, funded by the German Innovation Fund of the Federal Joint Committee (G-BA), is implementing this approach. As part of the project, parents of children and adolescents with an administrative ADHD diagnosis who were insured with a national statutory health insurance company (DAK-Gesundheit) were surveyed online about their child’s ADHD, utilisation of health care services, satisfaction with care and quality of life. The survey was based on the questionnaires used in the KiGGS study. In addition, a sub-sample of children and adolescents was clinically assessed using a guideline-based online diagnostics. The administrative data were then linked to the survey and clinical data at the person level. INTEGRATE-ADHD also included health economic issues.

The first results of INTEGRATE-ADHD are presented in articles published in the Journal of Health Monitoring in the third quarter of 2024. The article by Schlack et al. [2] describes the sometimes considerable discrepancies between administrative data and parental report of an ADHD diagnosis of the child, depending on various sociodemographic factors, and discusses various putative explanations. The article by Pfeifer et al. [3] examines which groups of medical specialists make the most frequent diagnoses of ADHD and the extent to which medical specialist diagnosis and utilisation of treatment providers are predictive of parental report of diagnosis. For this purpose, the administrative data and the online survey data were analysed in parallel. Using a diagnostic matrix to organise the complex diagnostic information collected through different assessor perspectives and methods, Hetzke et al. [4] describe the implementation of clinical ADHD diagnostics which had to be carried out online due to the SARS-CoV-2 pandemic. In addition, they highlight the challenges and opportunities of online diagnostics and point above to the considerable potential of telemedicine to extend the range of services available to children and adolescents with ADHD. In their contribution, Weyrich et al. [5] analyse the internal and interrater reliability of the ADHD section of the semi-structured diagnostic interview ILF-EXTERNAL (‘Interview-Leitfaden für Externale Störungen’) conducted with a parent via video chat. They were able to confirm both the good psychometric properties of the ILF-EXTERNAL and the high interrater reliability in the online setting, which ensures the quality of online diagnostics. Gilbert et al. [6] face up to the role of parental psychopathology and parental strain as risk factors on the one hand in their article for the quality of life of children and adolescents with ADHD. On the other hand, they discuss family climate, social support and utilisation of ADHD-related services as protective factors. In the last article, Hasemann et al. [7] use a matched control design employing propensity score matching to analyse the direct costs of service utilisation of children and adolescents with an incident ADHD diagnosis in the first year after diagnosis compared to an unaffected control group. Therefore, they considered the claims data of all children and adolescents in the DAK health insurance pool for the years 2018 to 2020.

At the time of publication of the articles in this issue of the Journal of Health Monitoring, the analyses from the project are not yet complete. In particular, data on clinical diagnosis and prescription and use of ADHD medication are currently being analysed. However, the results already show clear discrepancies between data sources and raise various questions about the health care of children and adolescents with ADHD. Answering these questions based on the results of INTEGRATE-ADHD will not only contribute to a better understanding and differentiation of various ADHD diagnostic data, but may also provide important impetus for optimising demand planning and resource management in the health care system. A society that understands how to effectively manage and support people with ADHD not only reduces individual suffering, but also achieves long-term economic and social benefits. The results of INTEGRATE-ADHD may contribute to making the care of people with mental disorders and ADHD in particular a task for the society as a whole.
